# Macroinvertebrate Community Responses and Recovery Mechanisms to Extreme Drought in Small Water Bodies of Eastern China

**DOI:** 10.3390/biology15100811

**Published:** 2026-05-21

**Authors:** Zhiqi Peng, Yili Zheng, Yaru Chen, Libo Han, Meng Wang, Beixin Wang

**Affiliations:** Department of Entomology, College of Plant Protection, Nanjing Agricultural University, Nanjing 210095, China; 2021202049@stu.njau.edu.cn (Z.P.); 2024102087@stu.njau.edu.cn (Y.Z.); 2023802226@stu.njau.edu.cn (Y.C.); 2023802225@stu.njau.edu.cn (L.H.); wmeng@njau.edu.cn (M.W.)

**Keywords:** community assembly, extreme drought, functional traits, macroinvertebrate, hydrological connectivity, ecological resilience, small water body

## Abstract

Extreme summer droughts increasingly threaten freshwater biodiversity in monsoonal regions, yet community responses within heterogeneous small water bodies (SWBs) remain poorly understood. This study evaluated how drying events influence macroinvertebrate taxonomic and functional composition across different SWB types and explored the mechanisms driving post-drought recovery. We sampled isolated ponds (IPs), stream-fed ponds (SFPs), pond-linked streams (PLSs), and non-pond-linked streams (NPLSs) in Eastern China during an extreme summer drought (2022) and a subsequent recovery year (2023). Ponds exhibited high resistance, maintaining stable taxonomic and functional richness. PLSs suffered substantial summer biodiversity declines but showed rapid post-drought recovery, possibly facilitated by spatial dispersal from nearby pond refuges. In contrast, NPLSs experienced severe, lasting biodiversity loss. Drought conditions drove overall community homogenization, with spatial dispersal playing a more important role in structuring assemblages than environmental filtering. Furthermore, functional trait analysis indicated that post-drought recovery was more closely associated with resilience-linked traits rather than resistance traits. These findings demonstrate that lateral connectivity within SWB networks buffers drought impacts by providing refugial support and enabling rapid recolonization. Preserving diverse, interconnected SWBs is a critical management strategy for maintaining metacommunity resilience under growing climatic volatility.

## Simple Summary

Freshwater ecosystems are increasingly threatened by extreme summer droughts driven by climate change. Small water bodies, such as ponds and streams, are particularly vulnerable, yet we know little about how the aquatic animals living in them cope with and recover from these drying events. Based on field surveys of macroinvertebrate communities in Eastern China before, during, and after an extreme drought, our study reveals that connected networks of water bodies are vital for survival. We found that ponds act as stable safe havens during dry periods, allowing aquatic insects to survive and later recolonize nearby streams when the water returns. Conversely, isolated streams suffer severe, long-lasting biological damage. Protecting and restoring connected freshwater habitats, rather than just isolated patches, is a highly effective conservation strategy to maintain aquatic biodiversity in the face of unpredictable global weather patterns.

## 1. Introduction

Drought is generally defined as an unpredictable event of unusually large and prolonged water scarcity, and this deficit causes a sufficient hydrological imbalance compared to the long-term average [1,2]. These hydrological changes, compounded by climate changes, are driving an unprecedent decline in freshwater biodiversity [3]. In recent years, the East Asian subtropical monsoon region, particularly the Yangtze River Basin in central and eastern China, has faced increasingly frequent and severe summer droughts [4], as exemplified by the extreme drought conditions of 2022, the most severe event recorded in the lower Yangtze region (including Nanjing) in nearly a century, which was likely exacerbated by climate change [5]. Despite the increasing regularity of such climatic extremes, how summer droughts affect freshwater biodiversity in East Asia remains poorly understood [6].

The responses of freshwater biodiversity to drought are complex, as they are shaped by multiple factors [7], one critical determinant being the type of water body [8]. Lotic ecosystems (i.e., streams or rivers) experience rapid declines in flow, increased temperature, reduced dissolved oxygen, and habitat fragmentation during drying, whereas lentic ecosystems (i.e., ponds or pools) primarily undergo gradual water-level reduction and thermal/chemical stratification [9,10]. These contrasting environmental changes impose different constraints on aquatic communities. Within this context, small water bodies (SWBs), which include many lentic, lotic and semi-lotic habitats, have gained increasing ecological recognition because their limited surface and volume, and strong hydrological connectivity make them especially sensitive to drying-driven environmental shifts [11,12]. Despite their importance for biodiversity conservation and ecosystem functioning, SWBs remain less studied than larger water bodies, leaving important gaps in our understanding of how drought-induced drying impacts these freshwater ecosystems.

Macroinvertebrates are widely used reliable indicators of drying impacts because they respond rapidly to altered habitat availability and water quality [13,14]. Drying can reduce taxonomic richness and selectively disadvantage sensitive taxa (e.g., Ephemeroptera, Plecoptera and Trichoptera), while also restructuring functional composition through the loss of flow-associated and cold-stenothermal taxa and changes in feeding modes [15,16,17,18]. However, taxonomic and functional responses may be decoupled: functional richness (FRic) often declines with the loss of key trait combinations, whereas functional redundancy (FRed) may increase transiently as assemblages become dominated by a subset of generalists, before declining under prolonged or repeated drying [19,20,21]. Post-drying trajectories depend on both resistance traits that enable in situ persistence (e.g., desiccation-resistant stages) and resilience traits that facilitate recolonization after rewetting (e.g., high dispersal ability, rapid development) [22]. Field and experimental studies commonly show that recolonization is initiated by taxa with high mobility and short generation times (e.g., chironomids and some mayflies and stoneflies), although the balance between compositional change and shifts in functional strategies varies among regions [23,24,25,26]. Despite extensive work in Mediterranean-climate rivers, evidence from monsoonal regions—particularly for SWBs in East Asia—remains limited [27,28].

Community assemblages of SWBs are expected to reflect both local environmental filtering and regional dispersal processes, with their relative importance varying across hydrological connectivity and spatial distance [29]. In highly connected, small-scale ecosystems, mass effects may play an important role, thereby enabling taxa to persist in environmentally suboptimal habitats due to the high level of dispersal from suitable habitats [30]. There is evidence suggesting that macroinvertebrates inhabiting drying mosaics may show stronger dispersal capacity than those in perennial stream ecosystems [31], making connectivity a key driver of community resilience during hydrological fluctuation. Under severe or prolonged drying, hydrological contraction alters environmental conditions (e.g., temperature, oxygen, chemistry), strengthening environmental filtering while simultaneously reducing spatial connectivity [32]; therefore, the relative contribution of environmental filtering to shaping community structure is likely to increase.

**Figure 1:**
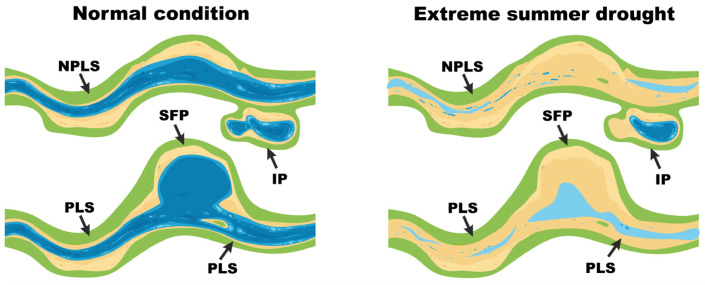
A conceptual diagram illustrating the four SWB types in ZNFP.

In China, SWBs (defined as having a catchment area of <0.1 km^2^) comprise a substantial part of inland water area and are particularly dense in parts of the lower Yangtze region (7.21 per km^−2^) [33]. Here, we focus on precipitation-fed SWBs (the exclusive SWB type in our study area) that can be categorized by physical and hydrological characteristics into four types: isolated ponds (IPs), stream-fed ponds (SFPs), pond-linked streams (PLSs) and non-pond-linked streams (NPLSs) (Figure 1). IPs are small, isolated, lentic and terrestrial SWBs formed by water accumulation in topographic depressions and exclusively fed by vertical precipitation. NPLSs are SWBs continuously flowing within an isolated channel, and the water source is rainfall and condensate collected from a valley. SFPs and PLSs function as dual components within integrated water networks characterized by both lentic and lotic water, where their up/downstream orientation is inconsequential. IPs and SFPs share the same water-fed mechanisms, so do PLSs and NPLSs, but high hydrological connectivity allows SFPs and PLSs to sustain water flow, mitigating complete desiccation during drought through reciprocal water supplementation. Located in the Yangtze River Basin—a core area affected by the 2022 drought—Nanjing City, Jiangsu Province, experienced its most severe drought in 72 years [34,35]. This drought provided an opportunity to examine how extreme drying events affect macroinvertebrate communities across these four SWB types.

The primary objective of this study was to evaluate how extreme drying events affect macroinvertebrate taxonomic and functional composition across different SWB types and to identify the mechanisms driving post-drying community reorganization. Specifically, we tested four hypotheses: (H_1_) community composition shift most strongly during peak summer drying, with the largest changes in NPLSs and the most in IPs; (H_2_) hydrologically connected habitats (SFPs and PLSs) retain higher local diversity (taxonomic richness and functional richness) and abundance during drought than IPs and NPLSs, consistent with buffering by connectivity; (H_3_) the relative contribution of environmental versus spatially structured processes to community composition varies seasonally, with drying expected to strengthen environmental control relative to normal conditions; (H_4_) in streams, post-drought reorganization is associated primarily with resilience-related trait modalities (e.g., dispersal and rapid life cycles), and trait shifts are more pronounced in PLSs than in NPLSs, owing to greater hydrological persistence and recolonization opportunities. To test these hypotheses, we capitalized on the extreme 2022 drought in the Yangtze River Basin—where Nanjing City experienced its most severe drought in 72 years [33,34]—as a natural experiment. We conducted seasonal field sampling (pre-drought spring, peak-drought summer, and post-drought winter) in 2022, along with follow-up sampling in streams in 2023, to examine macroinvertebrate communities across the four SWB types.

## 2. Materials and Methods

### 2.1. Study Area

**Figure 2:**
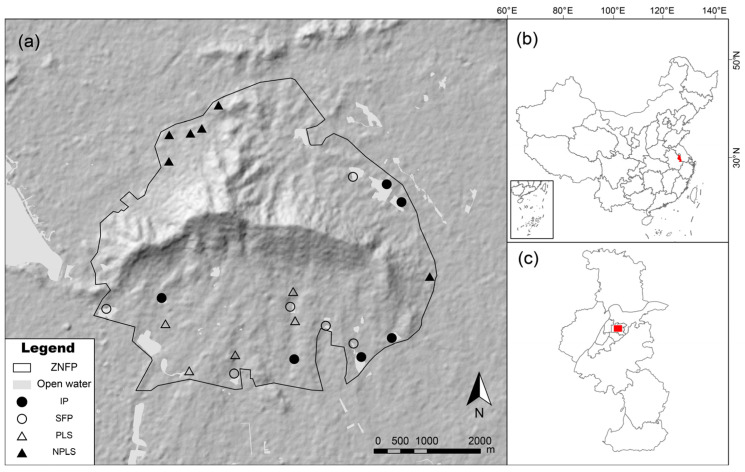
Location of (**a**) sampling sites in ZNFP; (**b**) the study area in Nanjing, China; and (**c**) ZNFP within Nanjing City.

Zijinshan National Forest Park (ZNFP) spans approximately 30.1 km^2^ in the central urban area of Nanjing City, Jiangsu Province, southern China (Figure 2). The landscape is predominantly hilly (maximum elevation 448.9 m) and underlain mainly by granite, gneiss, and sandstone [36]. The region has a subtropical monsoon climate with hot, humid summers and relatively cold, dry winters. As an isolated mountain ecosystem, ZNFP contains a dense network of SWBs that are exclusively fed by precipitation, with no evidence of groundwater inputs. Following preliminary field investigations, we purposefully selected 23 sampling sites representing all SWB types present within ZNFP. These sites lacked direct point-source anthropogenic pollution, allowing us to isolate the ecological impacts of drought from local water quality degradation.

### 2.2. Impact of the 2022 Drought

Since 1900, annual precipitation within ZNFP has remained relatively stable (long-term average: 1050.73 mm; range: 538.85–1561.07 mm, Supplementary Figure S1a), despite persistent warming trends associated with global climate change (Supplementary Figure S1b). In 2022, annual precipitation fell to 906.75 mm, representing a 13.7% deficit relative to the long-term average, whereas precipitation in 2023 (1250.81 mm) exceeded this baseline by 19.0%. The 2022 precipitation deficit culminated in an extreme summer drought in August, during which Nanjing experienced record-high temperatures (40.4 °C) and record-low precipitation (11.3 mm). Concurrently, sunshine duration was over 20% higher and precipitation more than 60% lower than the historical averages recorded since 1961 [37]. This extreme drought triggered pronounced shifts in the hydrological regime of ZNFP throughout 2022 (Supplementary Figure S1d) compared to the monthly precipitation baseline from 1900 to 2023 (Supplementary Figure S1c), whereas hydrological conditions in 2023 returned to comparatively normal levels (Supplementary Figure S1e). Consequently, water volumes in ZNFP ponds were substantially reduced, and stream discharge declined markedly or ceased entirely (Supplementary Figure S2).

In 2022, a total of 23 sites across four SWB types (IP, *n* = 6; SFP, *n* = 6; PLS, *n* = 5; and NPLS, *n* = 6) were sampled in April (spring), August (summer) and December (winter). During the peak of the extreme drought in the summer of 2022, a substantial proportion of the sampled sites experienced complete surface desiccation. Specifically, SWBs were entirely lost in 20% of PLSs and 83.3% of NPLSs (detailed hydrological statuses are provided in Supplementary Table S1).

In 2023, follow-up sampling was conducted exclusively in the streams (PLSs and NPLSs). During the summer drought of 2022, these lotic SWBs experienced the most severe drying event. Therefore, our primary objective was to evaluate the post-drought recolonization of these highly impacted assemblages. Consequently, ponds were not resampled in 2023. Our investigation into community reorganization focuses specifically on the stream SWBs.

### 2.3. Environmental Measurements

In addition to recording the coordinates using GPS positioning, we measured the width, depth, and velocity of streams at each site [38], as well as the surface area and depth of ponds. At each site, we collected a 200 mL water sample and stored it in the dark at 4 °C for subsequent laboratory analysis of total phosphorus (TP), total nitrogen (TN), ammonia nitrogen (NH_3_-N) and chemical oxygen demand (COD) following the Surface Water and Pollution Monitoring Technical Standards [39]. Additionally, water temperature (WT), electrical conductivity (EC), pH and dissolved oxygen (DO) were measured in situ by a portable meter (METTLER-TOLEDO SevenGo Duo^TM^, SG23 and Seven2Go^TM^, S4, Mettler-Toledo, Greifensee, Switzerland).

### 2.4. Macroinvertebrate Sampling and Identification

We sampled macroinvertebrates using a D-frame dip net (0.3 m width, 500 µm mesh). To comprehensively represent the local macroinvertebrate community, a proportional multi-habitat sampling approach was employed [40]. At each site, we collected ten sub-samples (0.3 m length each, totaling an area of 0.9 m^2^) in stream reaches or along the wadable areas of ponds. For streams, the ten sub-samples were allocated in proportion to the occurrence frequency of microhabitats (e.g., pools, riffles, leaf litter, water depth, substrate size, etc.) [41]. For ponds, the ten sub-samples were allocated in proportion to the occurrence frequency of microhabitats (e.g., submerged macrophytes, emergent macrophytes, floating macrophytes and open water) [42]. All ten sub-samples from each site were combined into a composite sample, preserved with 90% alcohol in the field, and then visually hand-picked with a 10 × amplifier in the laboratory. Most specimens were identified to the lowest possible taxonomic level (mainly species or genus), except Noteridae, Sisyridae and Belostomatidae (Supplementary Table S2) [43].

### 2.5. Functional Traits and Drought-Related Strategy

Following Usseglio-Polatera et al., we applied 10 functional traits including life history (e.g., life-cycle duration and adult life span), resilience or resistance potentials (e.g., dispersal, locomotion and armoring), and some physiological features (e.g., body size, feeding propensity and respiration type) to describe macroinvertebrate adaptation to disturbance (like drought, bad winters, or floods) [44]. We compiled a taxa-by-trait presence–absence (01) matrix mainly from published references [45,46] and online trait databases (http://www.freshwaterecology.info/index.php (accessed on 23 July 2023)). To maintain a binary matrix format, for taxa described by multiple modalities in a single category within these reference sources, we assigned a score of 1 to the modality with the highest affinity—representing the taxon’s primary functional trait—and 0 to all other modalities. Each taxon was coded according to its last aquatic life stage.

Drying adaptation traits can be categorized into either resilience (traits expected to facilitate recolonization after rewetting) or resistance (traits expected to facilitate persistence during drying event) traits, representing two distinct survival strategies that employ different physiological approaches [17]. For each trait, we identified resilience/resistance traits that do/do not promote survival in case of drought [47]. For instance, swimming, skating, and diving modalities can increase survival probability to drying events through strong dispersal abilities, while other movement habit modalities cannot. Similarly, taxa with well-protected arms and shelter enhance resistance through reducing water loss in living organisms, but other states do not. Conclusively, we classified all 35 trait modalities into four trait groups: resilience (RL), non-resilience (non-RL), resistance (RT), and non-resistance (non-RT) (Supplementary Table S3).

### 2.6. Data Analysis

#### 2.6.1. Community Composition

To address H_1_, patterns in community assemblage were visualized using non-metric multidimensional scaling (NMDS) with two dimensions. Ordinations were based on Bray–Curtis dissimilarities calculated from taxa abundance data. Sampling sites that were dry during investigation were retained and assigned zero abundance for all taxa to ensure their representation in the ordination space. Rare taxa were not excluded to preserve the sensitivity of the analysis to subtle community shifts [48].

Permutational multivariate analysis of variance (PERMANOVA, 999 permutations) was performed to test for differences in community composition. Prior to PERMANOVA, abundance data were transformed into a presence–absence matrix, and Raup–Crick dissimilarities were calculated. We chose the Raup–Crick metric over abundance-based Bray–Curtis coefficients for hypothesis testing to account for the extreme ecological conditions imposed by the drought. Complete desiccation at several sites during summer resulted in “all-zero” samples. Ecologically, a drying site represents a distinct habitat state rather than a mere sampling absence. Unlike Bray–Curtis, which handles all-zero samples poorly and can heavily skew multivariate outputs, the Raup–Crick metric robustly accommodates these extreme states. To further explore community recovery, pairwise PERMANOVA was conducted to compare assemblages between seasons, SWB types, and sampling years.

To identify specific macroinvertebrate taxa significantly associated with distinct SWB types and interannual periods, we performed an indicator species analysis (ISA). We utilized the group-equalized indicator value index (IndVal) to account for potentially unequal sample sizes across groups. The statistical significance of the indicator values for each taxon was evaluated using Monte Carlo random permutations (999 permutations), with the significance threshold set at *p* < 0.05.

#### 2.6.2. Calculation and Statistical Analysis of α-Diversity

We calculated TR and total abundance to quantify local taxonomic α-diversity. We applied FRic and FRed to represent local functional α-diversity based on a taxon–trait matrix and a sample–taxon matrix. FRic measures the number of unique trait combinations in the assemblages [49]. FRed quantifies the degree to which taxa in a community share similar function [50], so we thereby defined FRed as the degree to which a community is “saturated” with similar traits and calculated it as the ratio of Simpson diversity index to Rao’s quadratic entropy [51].

Exploring H_2_, we examined differences in α-diversity indices (TR, abundance, FRic and FRed) among SWB types across years using one-way analysis of variance (ANOVA) separately for each season. Prior to the analysis, all response variables were evaluated for normality using the Shapiro–Wilk test and for homogeneity of variances using Levene’s test. Data that violated these parametric assumptions were log(x + 1)-transformed to meet the requirements. When a significant overall ANOVA result was detected, post hoc multiple comparisons were performed using Fisher’s least significant difference (LSD) test to identify pairwise differences among SWB types. To adjust for multiple comparisons, the resulting *p*-values were corrected using the false discovery rate (FDR) method.

Similarly, we employed the same approach to examine the differences in environmental variables among different SWB types and seasons.

#### 2.6.3. Variation Partitioning

To test H_3_, we used Moran spectral randomization (MSR)-based variation partitioning. This analysis distinguished the relative contributions of environmental and spatial factors to taxonomic community composition across the three seasons of 2022. Prior to the analyses, biological abundance data were Hellinger-transformed, and environmental variables were log(x + 1)-transformed [38]. Taxonomic dissimilarity was calculated based on community data (sites × taxa abundances) using Bray–Curtis coefficients. Environmental distance was computed as the pairwise Euclidean distance between sites using the full set of normalized (mean = 0, SD = 1) environmental variables [52]. To avoid severe multicollinearity and model overfitting, we employed a forward selection procedure to identify the most parsimonious set of environmental predictors.

Spatial factors were derived using Moran’s eigenvector maps (MEMs) based on geographical distances between sites. Geographical distance between sites were calculated using the “Analysis Distance” tool in ArcGIS Map 10.8 software. We only used geographical Euclidean distance to represent spatial factors given that sampling sites exhibited negligible elevation gradients (Supplementary Table S1). To enable seasonal comparisons, we performed forward selection to identify the most influential spatial and environmental predictors, retaining the first four positive MEM eigenvectors and the first four environmental variables for each season. Total community variation was partitioned into four components: the pure effect of environmental variables ([E|S]), the pure effect of spatial factors ([S|E]), the joint effects of environment and spatial factors ([E∩S]), and unexplained variation [53].

To statistically evaluate differences in the relative importance of these three components, we extracted the MSR values for the [E|S], [S|E], and [E∩S]. The comparisons between the MSR values of pairwise components were performed separately for each season and SWB combination using Dunn’s post hoc tests. To control the false discovery rate across multiple pairwise tests, *p*-values were strictly adjusted using the Benjamini–Hochberg method.

#### 2.6.4. Shifts of Trait Modality During Stream Recovery

To address H_4_, we obtained a site × trait matrix by multiplying the site × taxa matrix (abundance) with the taxon × trait (presence–absence) matrix. The values in the site × trait represent the percentage of each trait modality within the community, with the sum of each trait category normalized to 100%. Using Mann–Whitney U tests, we identified trait modalities that showed significant changes in their relative proportions between 2022 and 2023 within either PLSs or NPLSs [47]. We used Sankey diagrams to relate trait modalities to PLSs or NPLSs and visualized the significant relationships and trait modalities with *p* < 0.05.

All analyses were conducted in R (version 4.0.3). We used the packages “vegan” [54] for NMDS and PERMANOVA, “FD” [55] for functional diversity indices, “agricolae” [56] for ANOVA and LSD tests, “rstatix” for Dunn’s post hoc tests, t-tests and *p*-value adjustments [57], “ade4” [58] for spatial analysis, “spdep” [59], “indicspecies” for ISA [60] and “adespatial” [61] for spatial dependence modeling and forward selection.

## 3. Results

### 3.1. Environmental Variables

**Figure 3:**
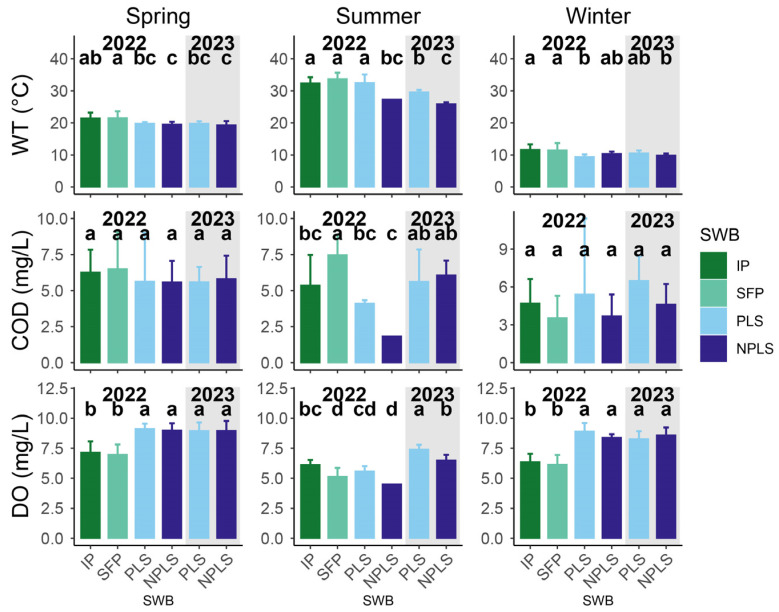
Average and standard deviation of physiochemical variables with significant seasonal or interannual differences across SWBs. The grey background denotes the sampling period in 2023, distinguishing it from 2022 (white background). Bars sharing no common lowercase letters are significantly different from one another within each season (Fisher’s LSD, *p* < 0.05).

In 2022, most measured environmental variables were generally similar between streams and ponds (Supplementary Figure S3), except DO, WT and COD. Streams showed significantly higher DO and COD but lower WT than ponds (Figure 3). Relative to 2023, streams in the summer of 2022 exhibited lower WT but higher COD and DO.

### 3.2. Taxonomic Composition and Seasonal Differences in Assemblage Structure

Across all samples, we collected 19,417 individuals representing 83 taxa across 45 families and 17 orders (Supplementary Table S2). Diptera (22 taxa) and Odonata (15 taxa) were the most taxon-rich orders. Fifty-nine taxa occurred in both ponds and streams; 11 taxa were exclusive to ponds and 13 to streams. The most abundant taxa included chironomids (*Polypedilum* sp., *Chironomus* sp.), mayflies (*Cloeon* sp., *Parafronurus youi*), shrimp (*Caridina pingi*) and snails (*Sinotaia quadrata*).

**Figure 4:**
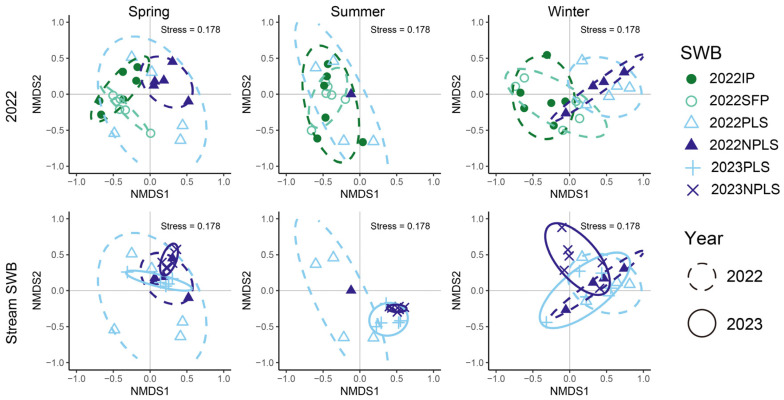
Community assemblages of three seasons based on Bray–Curtis distances.

In 2022, community assemblages differed considerably among seasons (PERMANOVA: spring vs. summer F = 2.804, *p* = 0.029; spring vs. winter F = 6.340, *p* < 0.001; summer vs. winter F = 5.622, *p* = 0.002) (Figure 4). In the spring, assemblages in NPLSs differed significantly from those in IPs (F = 9.820, *p* = 0.003) and SFPs (F = 10.191, *p* < 0.001). Specifically, the NPLS habitat was significantly indicated by eight taxa, most notably *Hydropsyche* sp. and *Antocha* sp., while *Sinotaia quadrate* and *Radix Auricularia* only appeared in ponds (ISA *p* < 0.001). These inter-SWB community differences became more pronounced during the summer and the winter. Beyond the persistent divergence between NPLSs and ponds, SFP assemblages in the summer differed considerably from those in both the PLSs (F = 4.148, *p* = 0.009) and NPLSs (F = 4.997, *p* = 0.013). No significant indicator taxa were identified between PLSs and NPLSs during the 2022 summer drought, and *Cricotopus* sp., *Polypedilum* sp. and *Simulium* sp. were the most notable taxa between SFPs and PLSs (ISA *p* < 0.01). In the winter, these observed spatial distinctions were maintained and further expanded by an additional, highly significant divergence between the IPs and PLSs (F = 53.019, *p* = 0.008). PLSs were significantly characterized by three indicator taxa, predominantly *Nothopsyche ruficollis* (ISA *p* < 0.01) and *Procambarus clarkii* (ISA *p* < 0.05), both of which occurred in 100% of the PLS samples.

For streams specifically, interannual comparisons revealed significant assemblage differences between 2022 and 2023 exclusively during summer (PLS F = 4.084, *p* = 0.013; NPLS F = 5.0287, *p* = 0.006); no significant interannual differences were detected in spring or winter. ISA across the summers of 2022 and 2023 identified 15 significant taxa characterizing specific SWB types (*p* < 0.05). NPLSs harbored the most distinct assemblage with six exclusive indicators (e.g., *Anisocentropus kawamurai*, IndVal = 0.982; *Polycentropus* sp., IndVal = 0.980). SFPs and IPs were uniquely characterized by three (e.g., *Rheocricotopus* sp.) and one (*Procloeon* sp.) taxa, respectively, whereas PLSs lacked exclusive indicators. Additionally, five taxa bridged habitat boundaries, most notably *Cricotopus* sp. (IndVal = 0.950), which strongly indicated both IPs and SFPs.

### 3.3. Taxonomic and Functional α-Diversity

**Figure 5:**
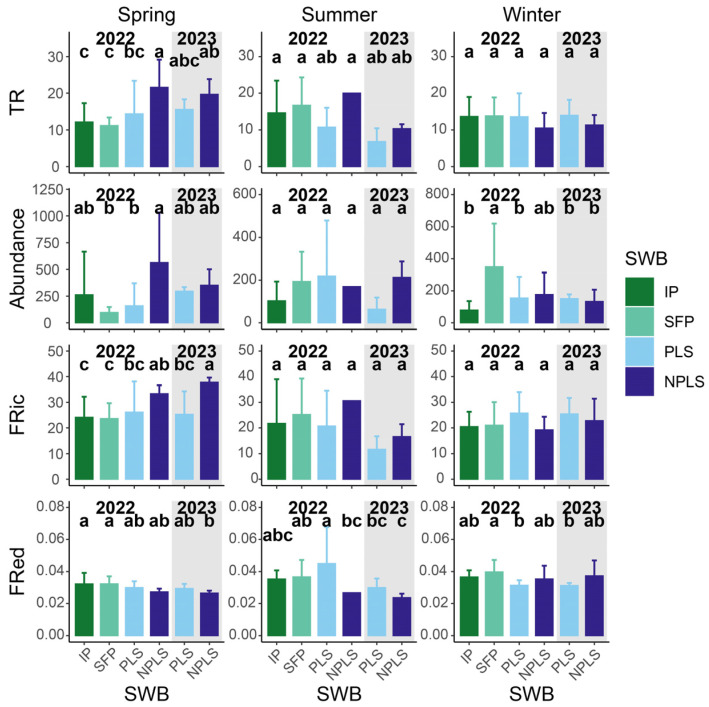
Average and standard deviation of TR, abundance FRic and FRed across SWBs. The grey background denotes the sampling period in 2023, distinguishing it from 2022 (white background). Bars sharing no common lowercase letters are significantly different from one another within each season (Fisher’s LSD, *p* < 0.05).

In 2022, α-diversity indices exhibited distinct seasonal contrasts among the SWB types (Figure 5). In spring, TR and FRic differed markedly between ponds and streams, with NPLSs generally exhibiting higher values than IPs, SFPs, and PLSs. In addition, abundance and FRed also varied among SWBs in the spring. Conversely, these spatial differences became less pronounced in the summer and were largely attenuated in the winter. Specifically, most indices showed limited statistical separation among SWB types in the summer of 2022. By the winter of 2022, TR, FRic, and FRed were broadly similar across SWBs, although the SFPs maintained a notably higher abundance.

Stream α-diversity showed distinct interannual changes between 2022 and 2023. In the spring of 2023, FRic still differed considerably between the PLSs and NPLSs while TR equalized and showed no significant difference. In contrast, summer and winter assemblages exhibited minimal interannual variation. Most diversity indices of summer and winter remained stable across both streams in both years.

### 3.4. Environmental and Spatial Contributions to Community Variation

**Figure 6:**
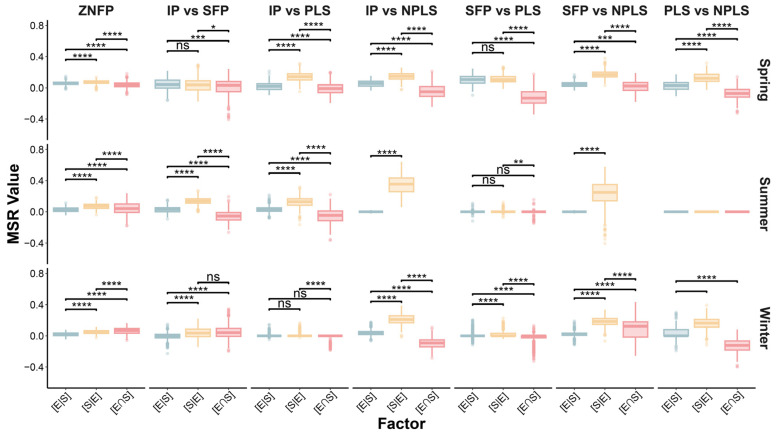
[E|S], [S|E] and [E∩S] in structuring community variation partitioning based on MSR. ns, not significant; *, *p* < 0.05; **, *p* < 0.01; ***, *p* < 0.001; ****, *p* < 0.0001.

The relative contributions of [E|S], [S|E], and [E∩S] to community composition remained consistent across the broader ZNFP region (Figure 6). Overall, [S|E] exerted a significantly greater influence on community composition than either [E|S] or [E∩S] across all seasons. This spatial dominance was particularly pronounced in all comparisons involving the NPLSs, most notably following drying events (the summer and winter of 2022). Interestingly, while the summer of 2022 exhibited the highest [S|E] contributions in NPLS-related comparisons, the explanatory powers of [E|S], [S|E], and [E∩S] between the SFPs and PLSs were near zero.

### 3.5. Trait Modalities Associated with Stream Recovery

**Figure 7:**
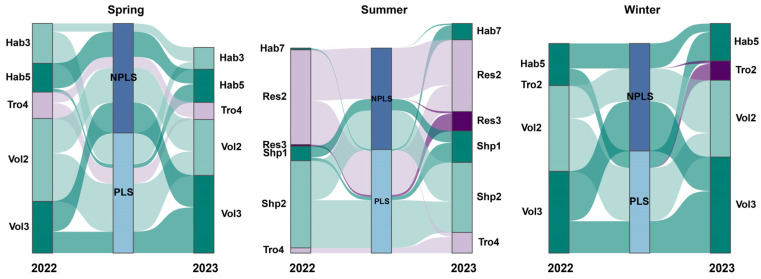
Significant linkages between NPLS and PLS and trait modalities. Only trait modalities with significant percentage changes are shown; all *p*-value are listed in Supplementary Table S4. Dark purple: RT; light purple: non-RT; dark green: RL; light green: non-RL.

Between 2022 and 2023, significant changes in trait modality proportions were concentrated in traits related to habit, trophic guild, respiration, life-cycle duration and body shape (Figure 7; Supplementary Table S4). Changes were more frequently associated with RL/non-RL than RT/non-RT group. Surprisingly, NPLSs appeared to function as more independent SWBs, their functional composition remained relatively stable, and the vast majority of traits exhibiting substantial interannual changes were exclusively associated with PLSs. The proportions of RT and RL groups increased in PLSs, whereas non-RT and non-RL groups decreased.

The most influential trait modality was bi-/multivoltine taxa (Vol3), which may lead to drought survival and subsequent recovery in PLSs, occurring at significantly higher proportions than in NPLSs. Spring and winter exhibited similar functional responses, characterized by increased proportions of swimming taxa (Hab5) and bi-/multivoltine taxa (Vol3). Additionally, the relative abundance of predators (Tro4) decreased during the spring, while collector–filterers (Tro2) increased during winter. In summer, shifts were more strongly associated with RT groups, including diving behavior (Hab7), streamlined body shape (Shp1) and specialized respiratory structures (valves, tracheae, and gas films; Res3).

## 4. Discussion

This study provides, to our knowledge, the first detailed assessment of how summer drying affects macroinvertebrates across different SWB types in East Asia. We sampled the seasons (spring, summer and winter) in 2022, spanning an extreme summer drought, and resampled streams in 2023 to evaluate how macroinvertebrate taxonomic and functional structure differed among SWB types. As predicted in H_1_, the extreme summer drought altered community structure, driving a transient convergence toward generalist-dominated assemblages across the network. H_2_ was also supported; PLSs maintained partial hydrological connectivity with adjacent SFPs and likely functioned as important refuges. Consequently, PLSs exhibited greater ecological resistance compared to hydrologically isolated NPLSs, which experienced complete desiccation and the extirpation of specialist taxa. Contrary to H_3_, variation partitioning showed that [S|E] explained more community variation than [E|S] across all seasons. Finally, functional trait analysis supported H_4_, and RL trait modalities were associated with stream recovery in spring and winter, whereas RT trait modalities were more closely associated with persistence during summer drought.

### 4.1. Assemblage Structure and α-Diversity Across SWB Types and Seasons

A reasonable interpretation is that freshwater faunas are strongly structured by habitat type, with streams and ponds typically supporting distinct species pools owing to differences in hydrological regime, substrate stability, and resource availability [62]. Under non-extreme conditions, NPLSs are therefore expected to be dominated by lotic specialists adapted to persistent flow environments, whereas PLSs, owing to their hydrological connectivity with ponds, are more likely to support generalist taxa capable of tolerating both lentic and lotic conditions. Our observations in the spring of 2022 were consistent with this expectation, as NPLSs supported several stream-specialist taxa (e.g., *Capnia zijinshana*, *Hydropsyche* sp. and *Lepidostoma flavum*), whereas PLSs and ponds contained a higher proportion of generalist and lentic-tolerant taxa (e.g., *Chironomus* sp., *Paracercion* sp. and *Sinotaia quadrate*).

However, this baseline spatial structure weakened markedly following the extreme summer drought of 2022. During summer, many NPLSs dried out completely, leading to the disappearance of several stream-specialist taxa and a marked simplification of community composition. The remaining assemblages were dominated by generalist taxa characterized by high dispersal capacity and broad ecological tolerances, including nine dipteran, two hemipteran, and four ephemeropteran or trichopteran taxa, as well as *Caridina pingi*. In contrast, PLSs retained fragmented flow conditions owing to partial water supply from adjacent ponds, allowing them to function as hydrological refugia during drying events. As a result, several taxa (e.g., *Dicrotendipes* sp., *Caenis* sp. and *Hydropsyche* sp.) absent from NPLSs during summer persisted in PLSs and SFPs and in some cases even increased in relative abundance. These patterns suggest that extreme drought can temporarily outweigh long-term habitat filtering, driving stream communities toward generalist-dominated assemblages.

The restructuring of community composition was mirrored by changes in α-diversity. TR and FRic declined in streams during the summer of 2022, with a more pronounced decrease in NPLSs than in PLSs. This contrast likely reflects hydrological differences, as pond–stream connectivity enabled PLSs to maintain partial flow and suitable habitat when NPLSs experienced complete drying. In ponds, by contrast, TR increased during drought, primarily through the proliferation of taxa with strong dispersal ability and rapid life cycles, such as Coleoptera (Dytiscidae and Haliplidae beetles), Hemiptera, and Chironomidae. Similar responses of Coleoptera assemblages to pond-level hydrological fluctuation have been reported previously [28]. Notably, many of these taxa were no longer observed following stream rewetting in winter, suggesting that their life-history strategies are adapted to monsoonal climates characterized by seasonal habitat shifts [63,64].

Functional diversity indices also suggested reduced ecosystem resilience under drought conditions. The tight coupling between FRic and TR is consistent with theoretical and empirical evidence demonstrating that FRic scales with TR [65]. Although FRed increased during summer in both streams and ponds, this pattern must be interpreted with caution. Rather than indicating true ecological redundancy, resilience, or a robust “insurance effect” for ecosystem stability, this transient peak in FRed likely reflects the metric’s behavior under severely reduced taxonomic richness. In streams, elevated FRed likely resulted from the loss of functionally unique specialist taxa, whereas in ponds, it was driven by the influx of ecologically similar generalists (e.g., *Dicrotendipes* sp., *Caenis* sp., and *Hydropsyche* sp.). Elevated FRed under such stress conditions may therefore indicate functional homogenization rather than enhanced ecosystem stability, particularly in systems with low overall functional uniqueness [46]. Therefore, this metric behavior probably indicates severe functional simplification and homogenization rather than enhanced ecosystem stability.

Furthermore, the interannual comparison between 2022 and 2023 suggests substantial resilience in these surface waterbody networks. While the extreme summer drought of 2022 drove significant compositional bottlenecks, the return of standard hydrological conditions in 2023 facilitated rapid community recovery [66]. As evidenced by our NMDS comparisons, the summer stream assemblages of 2023 differed significantly from those of the 2022 drought period yet exhibited comparable taxonomic and functional diversity across habitats. This interannual recovery suggests that, despite transient functional homogenization during severe desiccation, the preservation of regional species pools and intact dispersal pathways, particularly through the connectivity of pond–stream SWBs, enables the structural restoration of stream communities within a single annual cycle [67].

We emphasize that our inference of dispersal processes is based on observed changes in relative abundance and species turnover, combined with known trait syndromes such as dispersal capacity and life-history strategy, rather than on direct measurements of movement. Nonetheless, the contrasting responses of NPLSs and PLSs highlight the critical role of pond–stream connectivity in buffering stream communities against extreme drought. Maintaining or restoring natural connectivity between streams and ponds may therefore be essential for enhancing the resistance and recovery of stream-specialist macroinvertebrates under increasing climatic variability [68].

### 4.2. Importance of Dispersal Progress

Metacommunity recovery is jointly shaped by spatial processes and environmental filtering [69]; however, the relative importance of these mechanisms is largely mediated by the dispersal capacity of the taxa [70]. Consistent with this, our results indicated that [S|E] explained a larger proportion of community variation across all seasons in 2022, peaking during the summer. Dispersal processes are governed not solely by taxon-specific traits but also by the availability and proximity of source populations, which are critical for colonization and recolonization [71]. This concept is consistent with the near-zero explanatory power of [S|E] and [E|S] observed between SFP and PLS habitats during the summer. While this weak spatial and environmental structuring suggests high hydrological connectivity and associated mass effects that potentially homogenize community composition across these habitats [72,73], alternative explanations must be considered. The near-zero partitioning may simply reflect a low ecological signal relative to background variation. In highly fragmented drought environments, deterministic assembly is often overridden by strong stochasticity (e.g., ecological drift) or driven by unmeasured, localized micro-environmental variables and biotic interactions within confined refuges. Ultimately, this lack of explained variance underscores the highly complex and stochastic nature of community assembly under extreme hydrological stress. Conversely, landscape drying likely obstructs colonization pathways, potentially inducing severe dispersal limitations even when source populations are geographically proximate. Such constraints are characteristic of drought-impacted regions, where remnant waterbodies become largely disconnected [74]. Although ZNFP is spatially compact and Euclidean distances between SWBs are short, NPLSs appeared to function as effectively isolated islands. Their hydrological isolation and unique habitat characteristics were associated with strong spatial structuring, as evidenced by their consistently high [S|E] contributions. SFPs likely served as dry-season refuges; the homogenization between SFPs and PLSs suggests rapid movement of taxa into these refuges as surrounding habitats dried. Furthermore, the weak physicochemical discrepancy—presumably due to shared rainwater sourcing—may have facilitated the successful establishment of dispersing populations. However, the availability of a dry-season refuge does not universally guarantee positive outcomes for biodiversity. Several studies indicate that refuges may not significantly aid community recovery [21,75], potentially due to intensified biotic interactions, resource competition, and deteriorated water quality within confined refuge spaces. Mykrä et al. suggested that the influence of environmental filtering ([E|S]) typically increases at smaller spatial scales, whereas [S|E] and [E∩S] lose importance [76]. Conversely, our findings suggest that the 2022 drying events disrupted ecological connectivity, potentially causing this small-scale system to behave more like a set of isolated habitat islands and thereby elevating the importance of spatial constraints. Given the significant compositional differences between spring stream SWBs in 2022 and 2023, coupled with the lack of such differences in winter, we hypothesize that winter is a critical period for post-drought recovery and that the initial recovery cycle for ZNFP stream SWBs spans approximately one year.

Ultimately, drought reduced lateral connectivity among SWBs and likely promoted redistribution of strong dispersers from streams to ponds as stream habitats dried. Following rewetting, these taxa likely returned to streams. These seasonal changes support the hypothesis that dispersal-related spatial factors play a primary role in shaping macroinvertebrate composition, aligning with the overwhelming dominance of [S|E] over [E|S] observed throughout ZNFP. However, we emphasize that the causal pathways discussed here—particularly regarding dispersal-driven recovery and connectivity-mediated resilience—are inferred from observational field data. While our findings strongly align with these mechanisms, they should be interpreted as robust hypotheses rather than experimentally confirmed processes. To directly observe the exact ecological processes of community reorganization, future studies could reference the experimental frameworks of Aspin et al. to set up manipulative experiments, thereby rigorously testing these causal drivers under extreme drought [23].

### 4.3. Trait-Mediated Responses to Unpredictable Drought

Generally, RT and RL strategies exhibit strong responses along flow intermittency gradients, with taxa possessing these traits becoming increasingly prevalent in intermittent waterbodies [3,77]. Our findings indicate that traits associated with flexible survival strategies were especially important in PLSs, with habit-related categories displaying the greatest variability. For example, sprawling taxa (Hab3) represent the least dispersive group among RT strategists [78]. Consequently, these taxa experienced proportional declines in both PLSs and NPLSs, particularly in the latter. Flow cessation directly exposed these immobile taxa to desiccation, thereby increasing mortality. Conversely, swimming (Hab5) [79] and diving behaviors (Hab7) [80] likely facilitated the active search for remnant interstitial water as habitats dried. Furthermore, the increased prevalence of bi- and multivoltine taxa (Vol3) was accompanied by a concurrent decline in univoltine taxa (Vol2) during spring and winter, whereas no such shift occurred in summer. Increasing the number of generations per year can help populations stagger their life cycles to buffer against unpredictable drying events [81]. This mechanism aligns with observations related to thermal regimes [82], where rising water temperatures prompt certain stoneflies (e.g., *Leuctra nigra*) and mayflies (e.g., *Ephemerella danica*) to shift from semivoltine to univoltine life cycles [83]. Notably, shifts in respiration and body shape categories were entirely unique to the summer season. Reliance on specialized respiratory structures (e.g., valves, tracheae, and gas films; Res3) may reduce dependence on dissolved oxygen in the water column, mitigating the effects of drying [84]. Concurrently, crawling behaviors and streamlined body shapes (Shp1) allow taxa to resist flow in coarser substrates, facilitating active aquatic dispersal [85].

However, these pronounced functional shifts occurred predominantly within PLSs, whereas NPLSs only exhibited changes in movement mode preferences. The structural and functional alterations in PLSs were strongly mediated by landscape-scale environmental factors, including lateral connectivity, the proximity of refuges (a role fulfilled by SFPs in ZNFP), regional climatic characteristics, and the spatiotemporal changes in the drought. Crucially, our findings indicate that NPLSs function more as isolated habitats with limited ecological exchange with adjacent SWBs. The relative stability of their functional traits, combined with the accumulation of FRed and local population declines of specific taxa, has profoundly altered NPLS communities. This isolation may increase the vulnerability of these habitats under future climate change scenarios [18,86].

We also observed initial signs of ecological recovery within ZNFP. Our field evidence confirmed that following one year of secondary succession, communities in the streams of ZNFP had partially recovered by winter 2023. Some studies indicate that recovery from severe drying events can take two to three years or longer [87,88]. However, ecosystem responses vary significantly by disturbance type [89]; for example, impacts like bed dredging can allow macroinvertebrate fauna to regenerate relatively quickly during the subsequent period [90]. Conversely, prolonged or severe disturbances can trigger rapid transitions to novel ecosystem states [91,92]. We cannot assume that simpler or more isolated SWBs will recover as favorably as those in ZNFP. Therefore, protecting and monitoring macroinvertebrate biodiversity in SWBs should be a priority under ongoing climate change. The findings of this study underscore that preserving habitat heterogeneity and maintaining lateral connectivity are highly effective strategies for conserving freshwater fauna within SWBs.

## 5. Conclusions

Extreme summer drought significantly altered the macroinvertebrate communities across small water bodies, driving a temporary shift toward generalist-dominated assemblages. Ponds demonstrated high ecological resistance during the drought, maintaining stable taxonomic and functional richness. For streams, lateral connectivity played a decisive role in mitigating drought impacts. Pond-linked streams utilized adjacent ponds as hydrological refuges, which is associated with rapid recolonization and functional recovery. Conversely, non-pond-linked streams experienced complete desiccation, resulting in severe and lasting biodiversity loss due to their ecological isolation. Spatial dispersal processes primarily drove community assembly and post-drought recovery, outweighing local environmental filtering. This recovery may be more influenced by resilience-linked traits, such as swimming behaviors and multivoltine life cycles, rather than resistance strategies. These findings highlight the critical importance of preserving habitat heterogeneity and natural connectivity within freshwater networks. Conserving interconnected small water bodies is an essential management strategy to sustain metacommunity resilience against increasing climatic volatility.

## Data Availability

The data that support the findings of this study are openly available in the Environmental Data Initiative Portal at https://portal-s.edirepository.org/nis/mapbrowse?scope=edi&identifier=2291&revision=1 (updated on 2 March 2026).

## Supplementary Materials

The following supporting information can be downloaded at: https://www.mdpi.com/article/10.3390/biology15100811/s1, Figure S1: Annual (a) precipitation and (b) average temperature data calculated from 1900 to 2023 in Nanjing, eastern China. Also, the monthly average precipitation (c) from 1900 to 2023, (d) in 2022, (e) in 2023 are shown. The red and blue solid circles in (a) and (b) respectively represent the values of annual total precipitation and annual average temperature in 2022 and 2023; Figure S2: Habitat physical variables across SWBs; Figure S3: Physiochemical variables with non-significant seasonal or interannual differences across SWBs; Table S1: Characteristics of SWBs sampling sites in ZNFP during the sampling campaigns; Table S2: Relative composition (%) of macroinvertebrate sampled from ZNFP from 2022 to 2023; Table S3: Categorization of ten traits and 35 trait modalities into RT, nonRT, RL and nonRL groups; Table S4: Mann-Whitney U test results for trait modalities based on relative proportions between 2022 and 2023.
