# Supplementary material for: Macroinvertebrate Community Responses and Recovery Mechanisms to Extreme Drought in Small Water Bodies of Eastern China
Source: Biology (Basel). 2026 May 21;15(10):811. doi: 10.3390/biology15100811 (PMC13203084; doi:10.3390/biology15100811)
Supplement: Supplementary file 1 [file biology-15-00811-s001.zip › biology-4318023-supplementary.pdf]

Supplementary Figure

Supplementary Figure S1

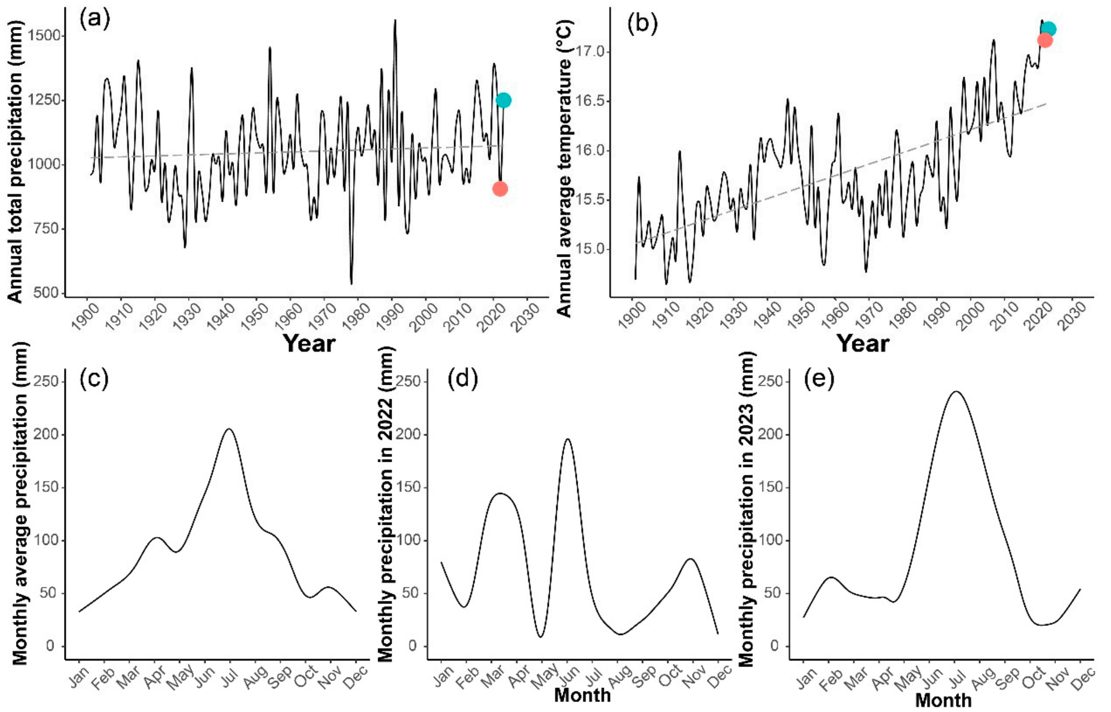

Supplementary Figure S1 Annual (a) precipitation and (b) average temperature data calculated from 1900 to 2023 in Nanjing, eastern China. Also, the monthly average precipitation (c) from 1900 to 2023, (d) in 2022, (e) in 2023 are shown. The red and blue solid circles in (a) and (b) respectively represent the values of annual total precipitation and annual average temperature in 2022 and 2023. The dashed gray lines in (a) and (b) represent the trends of annual total precipitation and annual average temperature from 1900 to 2023, respectively. Precipitation data was collected from https://cds.climate.copernicus.eu/cdsapp#!/dataset/reanalysis-era5-land-monthly-means?tab=overview, precipitation data was collected from https://data.tpdc.ac.cn/zh-hans/data/71ab4677-b66c-4fd1-a004-b2a541c4d5bf.

Supplementary Figure S2

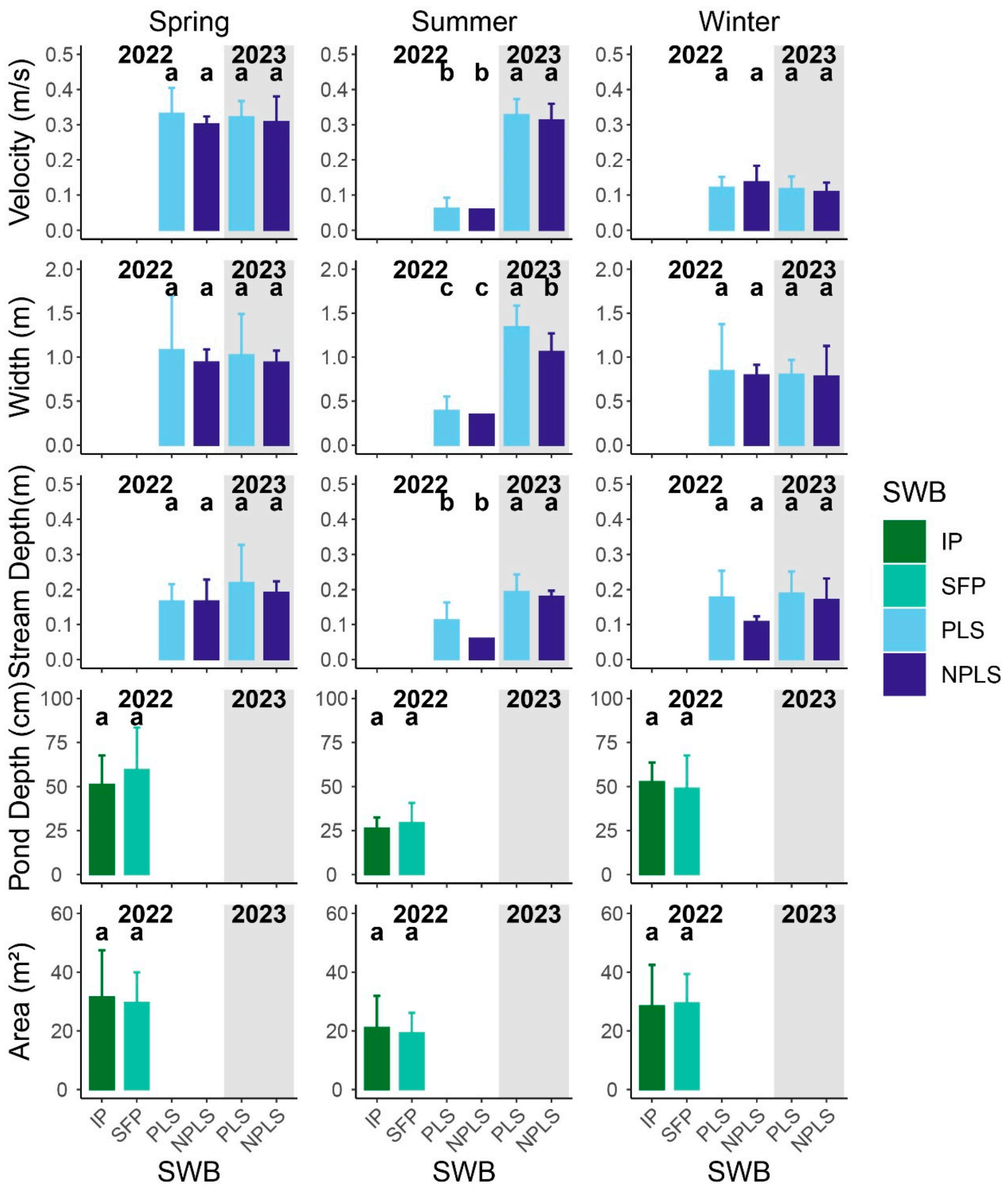

Supplementary Figure S2 Habitat physical variables across SWBs.

Supplementary Figure S3

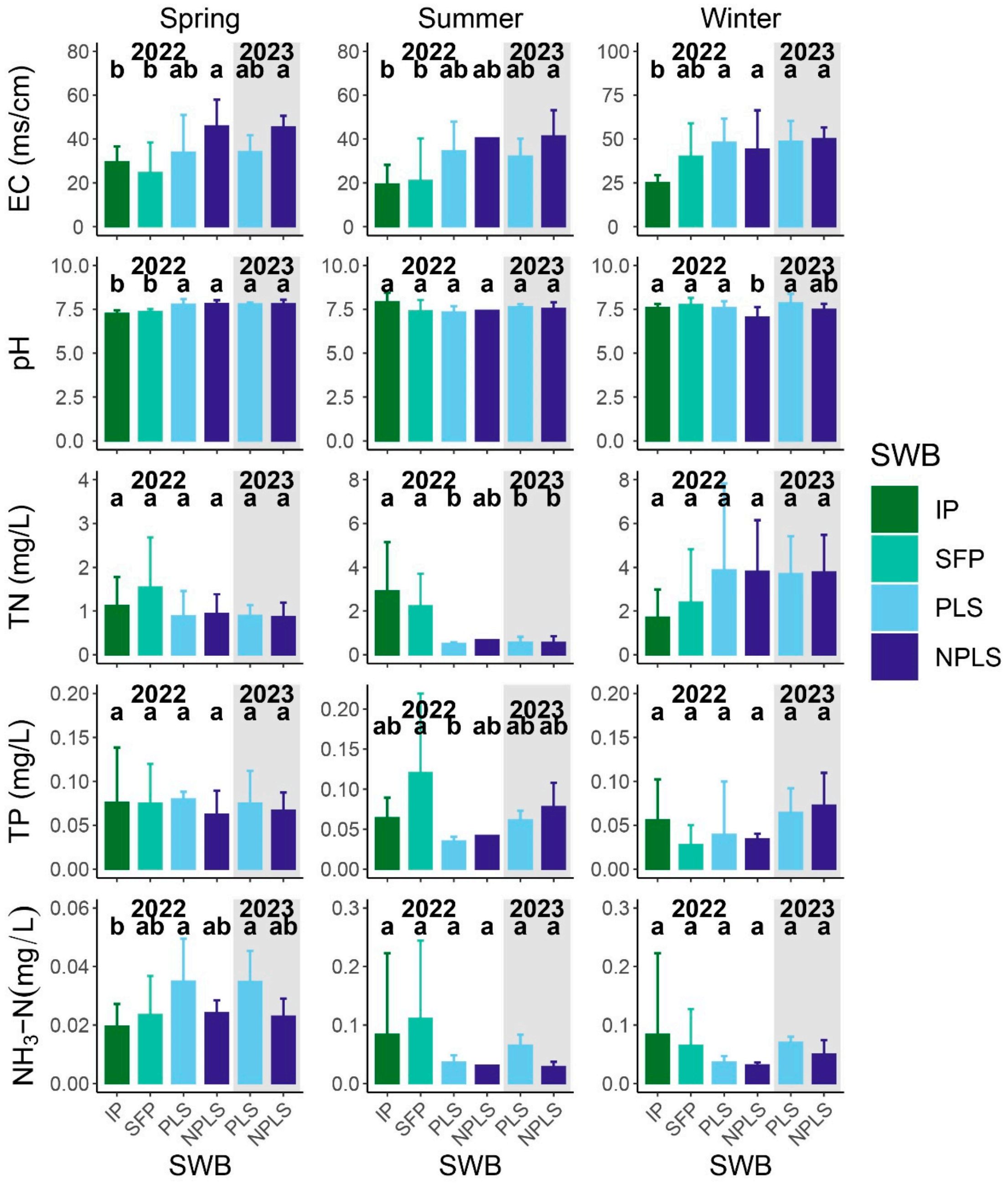

Supplementary Figure S3 Physiochemical variables with non-significant seasonal or interannual differences across SWBs.

Supplementary Table

Supplementary Table S1

Supplementary Table S1 Characteristics of small water bodies (SWBs) sampling sites in Zijinshan National Forest Park (ZNFP) during the sampling campaigns. Considering that not all water bodies have accurate names, we use iconic landmarks near some unnamed bodies of water to instead.

Site	Altitude	SWB	2023			2024		
			Spring	Summer	Winter	Spring	Summer	Winter
Site1	34	IP	W	W	W	-	-	-
Site2	34	IP	W	W	W	-	-	-
Site3	41	SFP	W	W	W	-	-	-
Site4	27	IP	W	W	W	-	-	-
Site5	48	SFP	W	W	W	-	-	-
Site6	53	SFP	W	W	W	-	-	-
Site7	19	SFP	W	W	W	-	-	-
Site8	34	PLS	W	D	W	W	W	W
Site9	24	PLS	W	W	W	W	W	W
Site10	18	SFP	W	W	W	-	-	-
Site11	21	PLS	W	W	W	W	W	W
Site12	31	IP	W	W	W	-	-	-
Site13	52	SFP	W	W	W	-	-	-
Site14	49	PLS	W	W	W	W	W	W
Site15	70	PLS	W	W	W	W	W	W
Site16	35	NPLS	W	D	D	W	W	W
Site17	54	NPLS	W	W	W	W	W	W
Site18	59	NPLS	D	D	W	W	W	W
Site19	36	IP	W	W	W	-	-	-
Site20	51	NPLS	W	D	W	W	W	W
Site21	45	NPLS	W	D	D	W	W	W
Site22	59	NPLS	W	D	W	W	W	W
Site23	36	IP	W	W	W	-	-	-

Note: IP: independent pond, SFP: stream-fed pond, PLS: pond-linked stream, NPLS: non-pond-linked streams, D: dried SWBs, W: wet SWBs, -: no collection.

Supplementary Table S2 Supplementary Table S2 Relative composition (%) of macroinvertebrate sampled from ZNFP from 2022 to 2023. Depth of shade indicates proportional dominance: white = 0, light = 0.1%–1%, medium = 1%–5%, medium-dark = 5%–20%, darkest = >20% relative composition per site. Grey represents no collection.

Phylum	Class	Order	Family	Genus	Taxon		IP						SFP						PLS						NPLS					
							2022			2023			2022			2023			2022			2023			2022			2023		
							Spr	Sum	Win	Spr	Sum	Win	Spr	Sum	Win	Spr	Sum	Win	Spr	Sum	Win	Spr	Sum	Win	Spr	Sum	Win	Spr	Sum	Win
Arthropoda	Insecta	Coleoptera	Dytiscidae	*Platambus*	*Platambus schillhammeri*	sp1																								
				Sandracottus	*Sandracottus hunteri*	sp2																								
			Elmidae	*Ampumixis*	*Ampumixis* sp.	sp3																								
			Haliplidae	*Haliplus*	*Haliplus* sp.	sp4																								
				Peltodytes	*Peltodytes* sp.	sp5																								
			Psephenidae	*Eubrianax*	*Eubrianax* sp.	sp6																								
			Scirtidae	*Scirtes*	*Scirtes* sp.	sp7																								
		Diptera	Ceratopogonidae	*Ceratopogonus*	*Ceratopogonus* sp.	sp8																								
			Chironomidae	*Acricotopus*	*Acricotopus* sp.	sp9																								
				Chaetocaldius	*Chaetocaldius* sp.	sp10																								
				Chironomus	*Chironomus* sp.	sp11																								
				Corynoneura	*Corynoneura* sp.	sp12																								
				Cricotopus	*Cricotopus* sp.	sp13																								
				Dicrotendipes	*Dicrotendipes* sp.	sp14																								
				Epoicocladius	*Epoicocladius* sp.	sp15																								
				Eukiefferiella	*Eukiefferiella* sp.	sp16																								
				Macropelopia	*Macropelopia* sp.	sp17																								
				Meropelopia	*Meropelopia* sp.	sp18																								

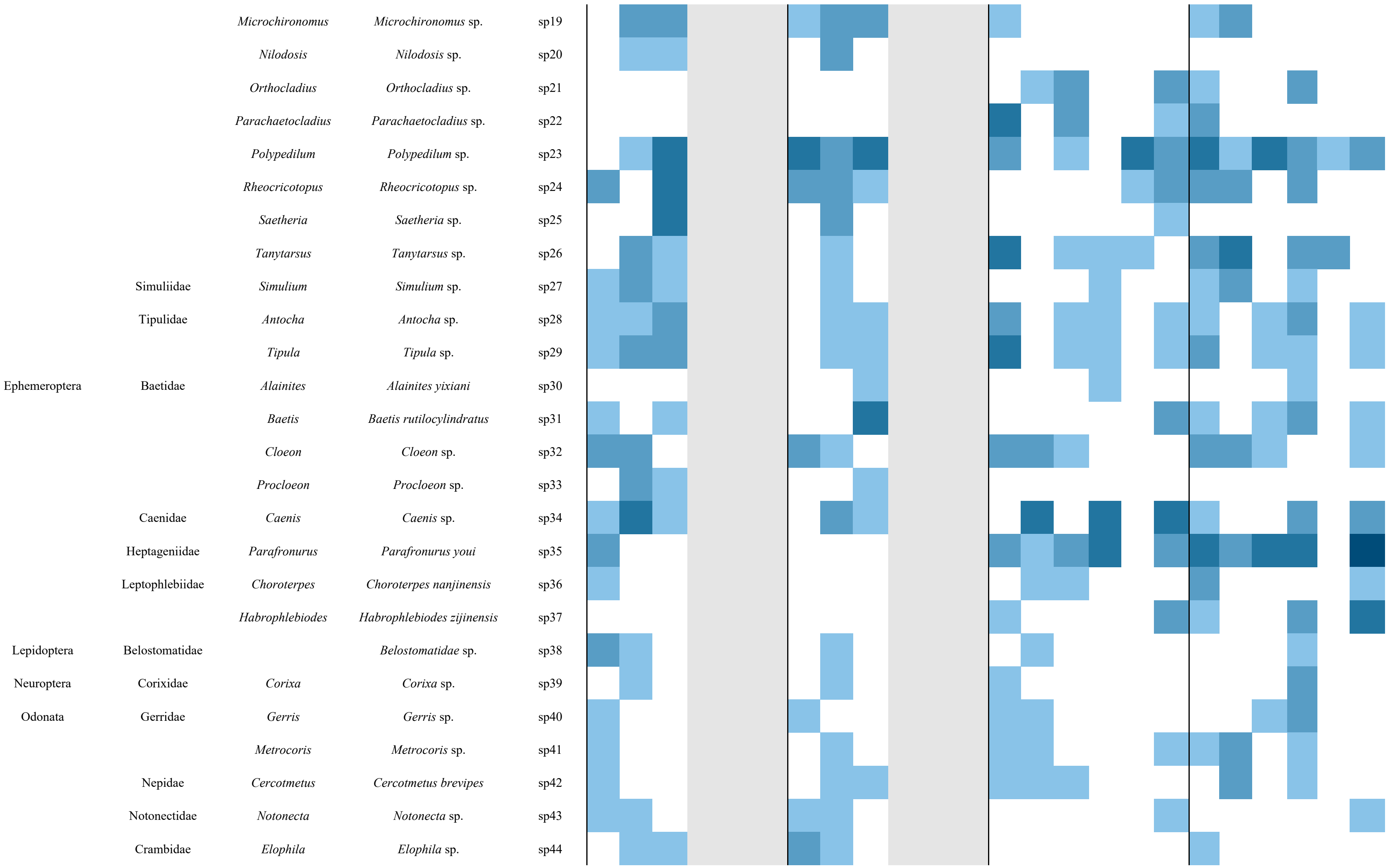
Microchironomus Microchironomus sp. sp19
Nilodosis Nilodosis sp. sp20
Orthocladius Orthocladius sp. sp21
Parachaetocladius Parachaetocladius sp. sp22
Polypedilum Polypedilum sp. sp23
Rheocricotopus Rheocricotopus sp. sp24
Saetheria Saetheria sp. sp25
Tanytarsus Tanytarsus sp. sp26
Simuliidae Simulium Simulium sp. sp27
Tipulidae Antocha Antocha sp. sp28
Tipula Tipula sp. sp29
Ephemeroptera Baetidae Alainites Alainites yixiani sp30
Baetis Baetis rutilocylindratus sp31
Cloeon Cloeon sp. sp32
Procloeon Procloeon sp. sp33
Caenidae Caenis Caenis sp. sp34
Heptageniidae Parafronurus Parafronurus youi sp35
Leptophlebiidae Choroterpes Choroterpes nanjinensis sp36
Habrophlebiodes Habrophlebiodes zijinensis sp37
Lepidoptera Belostomatidae Belostomatidae sp. sp38
Neuroptera Corixidae Corixa Corixa sp. sp39
Odonata Gerridae Gerris Gerris sp. sp40
Metrocoris Metrocoris sp. sp41
Nepidae Cercotmetus Cercotmetus brevipes sp42
Notonectidae Notonecta Notonecta sp. sp43
Crambidae Elophila Elophila sp. sp44

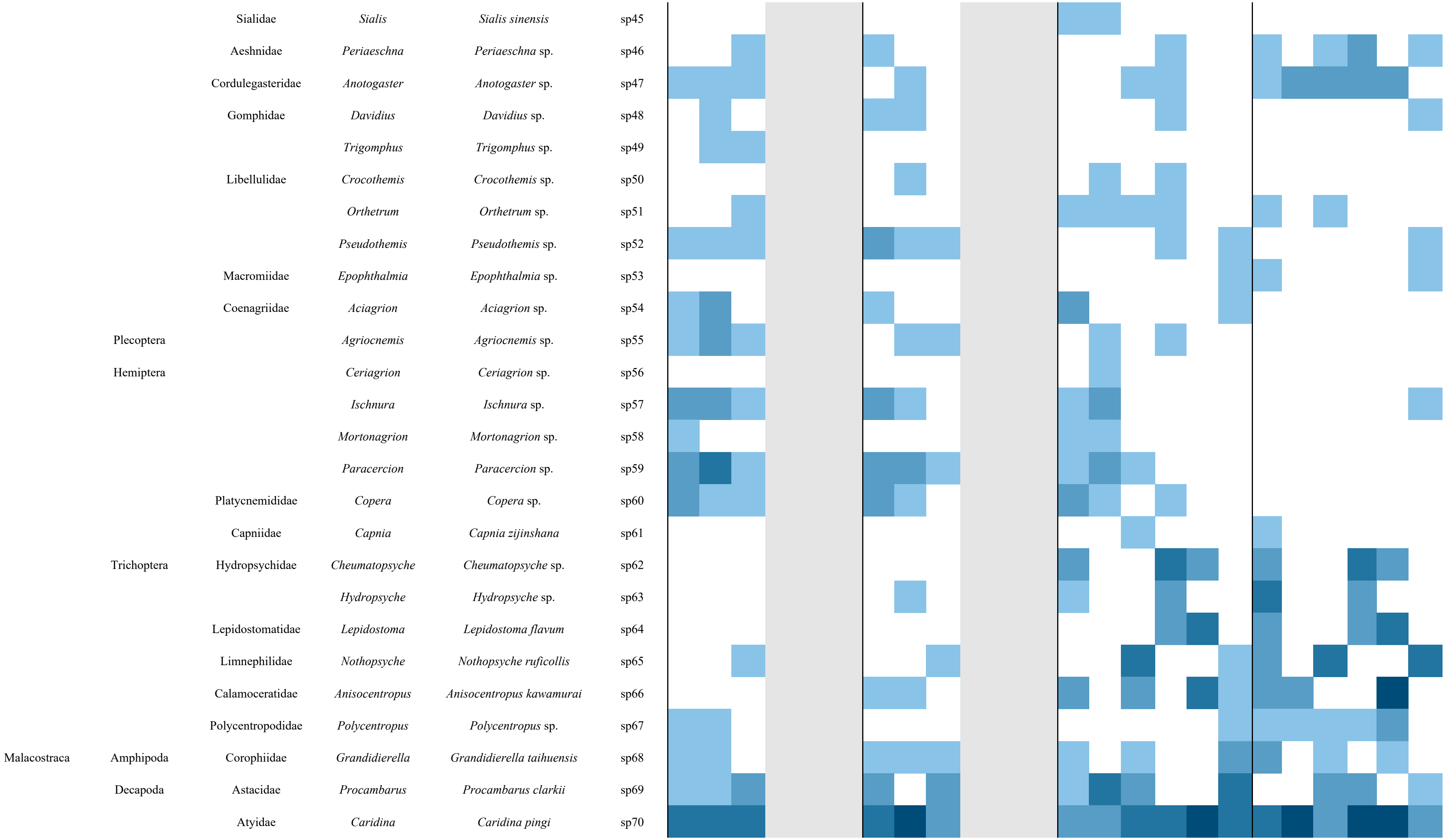

Sialidae Sialis Sialis sinensis sp45
Aeshnidae Periaeschna Periaeschna sp. sp46
Cordulegasteridae Anotogaster Anotogaster sp. sp47
Gomphidae Davidius Davidius sp. sp48
Trigomphus Trigomphus sp. sp49
Libellulidae Crocothemis Crocothemis sp. sp50
Orthetrum Orthetrum sp. sp51
Pseudothemis Pseudothemis sp. sp52
Macromiidae Epophthalmia Epophthalmia sp. sp53
Coenagriidae Aciagrion Aciagrion sp. sp54
Plecoptera Agriocnemis Agriocnemis sp. sp55
Hemiptera Ceriagrion Ceriagrion sp. sp56
Ischnura Ischnura sp. sp57
Mortonagrion Mortonagrion sp. sp58
Paracercion Paracercion sp. sp59
Platycnemididae Copera Copera sp. sp60
Capniidae Capnia Capnia zijinshana sp61
Trichoptera Hydropsychidae Cheumatopsyche Cheumatopsyche sp. sp62
Hydropsyche Hydropsyche sp. sp63
Lepidostomatidae Lepidostoma Lepidostoma flavum sp64
Limnephilidae Nothopsyche Nothopsyche ruficollis sp65
Calamoceratidae Anisocentropus Anisocentropus kawamurai sp66
Polycentropodidae Polycentropus Polycentropus sp. sp67
Malacostraca Amphipoda Corophiidae Grandidierella Grandidierella taihuensis sp68
Decapoda Astacidae Procambarus Procambarus clarkii sp69
Atyidae Caridina Caridina pingi sp70

			Palaemonidae	*Macrobrachium*	*Macrobrachium nipponense*	sp71
				Palaemon	*Palaemon* sp.	sp72
		Isopoda	Asellidae	*Asellus*	*Asellus* sp.	sp73
Mollusca	Gastropoda	Mesogastropoda	Ampullariidae	*Pomacea*	*Pomacea canaliculata*	sp74
			Bithyniidae	*Parafossarulus*	*Parafossarulus striatulus*	sp75
			Viviparidae	*Sinotaia*	*Sinotaia quadrate*	sp76
		Basommatophora	Lymnaeidae	*Radix*	*Radix auricularia*	sp77
			Planorbidae	*Hippeutis*	*Hippeutis cantori*	sp78
			Physidae	*Physa*	*Physa acuta*	sp79
	Bivalvia	Veneroida	Corbiculidae	*Corbicula*	*Corbicula fluminea*	sp80
Annelida	Clitellata	Haplotaxida	Naididae	*Limnodrilus*	*Limnodrilus* sp.	sp81
		Rhynchobdellida	Glossiphoniidae	*Glossiphonia*	*Glossiphonia lata*	sp82
		Arhynchobdellida	Erpobdellidae	*Erpobdella*	*Erpobdella octocilata*	sp83

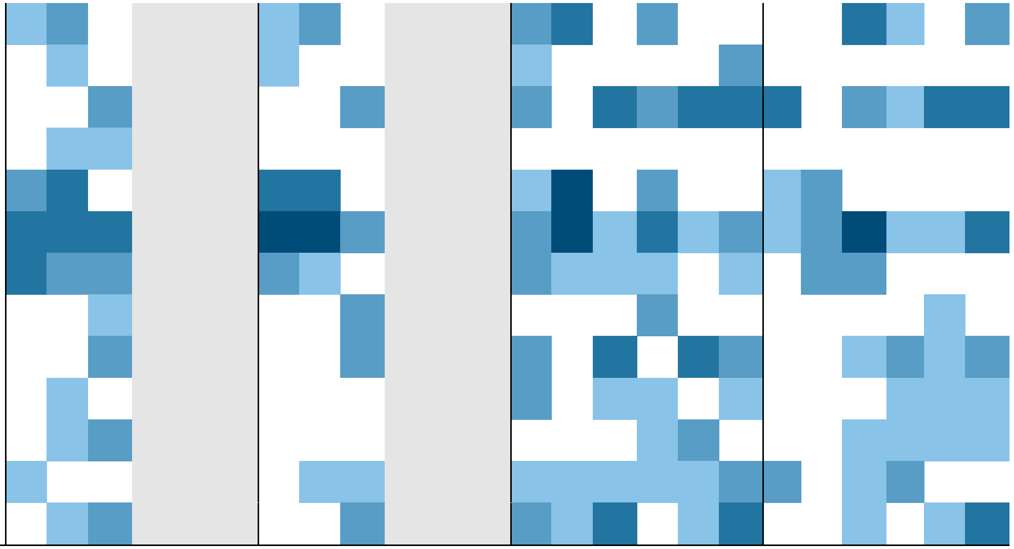

Note: Spr: spring, Sum: summer, Win: winter.

Supplementary Table S3 Supplementary Table S3 Categorization of ten traits and 35 trait modalities into resistance (RT), non-resistance (nonRT), resilience (RL) and non-resilience (nonRL) groups.

Trait categories	Trait modalities	Code	Group
Habit	Burrowers	Hab1	nonRL
	Climbers	Hab2	nonRL
	Sprawlers	Hab3	nonRL
	Clingers	Hab4	nonRL
	Swimmers	Hab5	RL
	Skaters	Hab6	RL
	Divers	Hab7	RL
Trophic habit	Collector-gatherer	Tro1	RT
	Collector-filterer	Tro2	RT
	Herbivore (scraper, piercer, and shedder)	Tro3	RT
	Predator (piercer and engulfer)	Tro4	nonRT
	Shredder (detritivore)	Tro5	RT
Respiration	Tegument	Res1	nonRT
	Gills	Res2	nonRT
	Valve, trachea, gas film	Res3	RT
Shelter	None	She1	nonRT
	Built webs	She2	RT
	Fine-grained sediments (e.g., sand, wood)	She3	RT
	Leaves	She4	RT
Life cycle duration	Semivoltine	Vol1	nonRL
	Univoltine	Vol2	nonRL
	Bi- & multi-voltine	Vol3	RL
Adult life span	Very short(< 1 week)	Life1	nonRT
	Short(1 - 4 week)	Life2	nonRT
	Long(> 1 month)	Life3	RT
Maximal potential size	Small(< 9 mm)	Size1	RT
	Medium(9 - 16 mm)	Size2	nonRT
	Large(> 16 mm)	Size3	nonRT
Flight performance	Weak	Fly1	nonRL
	Strong	Fly2	RL
Armoring	None (soft-bodied forms)	Arm1	nonRT
	Poor (heavily sclerotized)	Arm2	RT
	Good (well protected)	Arm3	RT
Body shape	Streamlined	Shp1	RL
	Not streamlined	Shp2	nonRL

Supplementary Table S4 Supplementary Table S4 Mann-Whitney U test results for trait modalities based on relative proportions between 2022 and 2023.

Season	Strategy	Code	Trait modalities	PLS *p* value	NPLS *p* value	Displayed on Fig. 5.
Spring	nonRL	Hab1	Burrowers	0.421	0.329	
Spring	nonRL	Hab2	Climbers	0.398	0.052	
Spring	nonRL	Hab3	Sprawlers	**0.032**	**0.009**	yes
Spring	nonRL	Hab4	Clingers	0.421	0.792	
Spring	RL	Hab5	Swimmers	**0.036**	0.126	yes
Spring	RL	Hab6	Skaters	NA	1.000	
Spring	RL	Hab7	Divers	1.000	0.082	
Spring	RT	Tro1	Collector-gatherer	0.841	0.792	
Spring	RT	Tro2	Collector-filterer	0.095	0.662	
Spring	RT	Tro3	Herbivore (scraper, piercer, and shedder)	1.000	1.000	
Spring	nonRT	Tro4	Predator (piercer and engulfer)	**0.016**	0.329	yes
Spring	RT	Tro5	Shredder (detritivore)	0.421	0.247	
Spring	nonRT	Res1	Tegument	0.056	0.931	
Spring	nonRT	Res2	Gills	0.056	0.247	
Spring	RT	Res3	Valve, trachea, gas film	0.666	0.082	
Spring	nonRT	She1	None	0.222	0.126	
Spring	RT	She2	Built webs	0.424	0.562	
Spring	RT	She3	Fine-grained sediments (e.g., sand, wood)	0.690	0.126	
Spring	RT	She4	Leaves	0.346	0.583	
Spring	nonRL	Vol1	Semivoltine	0.914	0.662	
Spring	nonRL	Vol2	Univoltine	**0.016**	0.662	yes
Spring	RL	Vol3	Bi- & multi-voltine	**0.016**	0.931	yes
Spring	nonRT	Life1	Very short (< 1 week)	0.095	0.177	
Spring	nonRT	Life2	Short (1 - 4 week)	0.421	0.126	
Spring	RT	Life3	Long (> 1 month)	0.421	0.429	
Spring	RT	Size1	Small (< 9 mm)	0.548	0.177	
Spring	nonRT	Size2	Medium (9 - 16 mm)	1.000	0.537	
Spring	nonRT	Size3	Large (> 16 mm)	0.548	0.662	
Spring	nonRL	Fly1	Weak	0.548	0.537	
Spring	RL	Fly2	Strong	0.548	0.537	
Spring	nonRT	Arm1	None (soft-bodied forms)	0.421	0.329	
Spring	RT	Arm2	Poor (heavily sclerotized)	0.222	0.177	
Spring	RT	Arm3	Good (well protected)	1.000	0.126	
Spring	RL	Shp1	Streamlined	0.548	0.792	
Spring	nonRL	Shp2	Not streamlined	0.548	0.792	
Summer	nonRL	Hab1	Burrowers	1.000	0.237	
Summer	nonRL	Hab2	Climbers	0.901	0.144	

Summer	nonRL	Hab3	Sprawlers	0.063	0.661	
Summer	nonRL	Hab4	Clingers	0.111	0.144	
Summer	RL	Hab5	Swimmers	0.556	1.000	
Summer	RL	Hab6	Skaters	0.131	NA	
Summer	RL	Hab7	Divers	**0.019**	0.237	yes
Summer	RT	Tro1	Collector-gatherer	0.730	0.144	
Summer	RT	Tro2	Collector-filterer	0.190	0.144	
Summer	RT	Tro3	Herbivore (scraper, piercer, and shedder)	0.286	0.144	
Summer	nonRT	Tro4	Predator (piercer and engulfer)	**0.032**	0.144	yes
Summer	RT	Tro5	Shredder (detritivore)	0.556	0.144	
Summer	nonRT	Res1	Tegument	1.000	0.144	
Summer	nonRT	Res2	Gills	**0.016**	0.144	yes
Summer	RT	Res3	Valve, trachea, gas film	**0.016**	0.144	yes
Summer	nonRT	She1	None	0.905	0.144	
Summer	RT	She2	Built webs	0.371	0.144	
Summer	RT	She3	Fine-grained sediments (e.g., sand, wood)	0.556	0.237	
Summer	RT	She4	Leaves	0.171	0.144	
Summer	nonRL	Vol1	Semivoltine	0.190	0.144	
Summer	nonRL	Vol2	Univoltine	1.000	0.661	
Summer	RL	Vol3	Bi- & multi-voltine	0.556	0.661	
Summer	nonRT	Life1	Very short (< 1 week)	0.556	0.144	
Summer	nonRT	Life2	Short (1 - 4 week)	0.712	0.144	
Summer	RT	Life3	Long (> 1 month)	0.556	0.144	
Summer	RT	Size1	Small (< 9 mm)	0.111	0.144	
Summer	nonRT	Size2	Medium (9 - 16 mm)	0.111	1.000	
Summer	nonRT	Size3	Large (> 16 mm)	0.063	0.140	
Summer	nonRL	Fly1	Weak	0.063	0.144	
Summer	RL	Fly2	Strong	0.063	0.144	
Summer	nonRT	Arm1	None (soft-bodied forms)	0.413	0.144	
Summer	RT	Arm2	Poor (heavily sclerotized)	1.000	0.144	
Summer	RT	Arm3	Good (well protected)	1.000	0.144	
Summer	RL	Shp1	Streamlined	**0.016**	1.000	yes
Summer	nonRL	Shp2	Not streamlined	**0.016**	1.000	yes
Winter	nonRL	Hab1	Burrowers	0.310	0.914	
Winter	nonRL	Hab2	Climbers	NA	0.904	
Winter	nonRL	Hab3	Sprawlers	0.841	0.914	
Winter	nonRL	Hab4	Clingers	0.056	0.352	
Winter	RL	Hab5	Swimmers	**0.310**	**0.038**	yes
Winter	RL	Hab6	Skaters	1.000	NA	
Winter	RL	Hab7	Divers	1.000	0.694	
Winter	RT	Tro1	Collector-gatherer	0.095	0.914	
Winter	RT	Tro2	Collector-filterer	**0.010**	0.149	yes

Winter	RT	Tro3	Herbivore (scraper, piercer, and shedder)	1.000	0.669	
Winter	nonRT	Tro4	Predator (piercer and engulfer)	0.310	0.914	
Winter	RT	Tro5	Shredder (detritivore)	0.548	0.171	
Winter	nonRT	Res1	Tegument	0.151	0.762	
Winter	nonRT	Res2	Gills	0.056	0.762	
Winter	RT	Res3	Valve, trachea, gas film	1.000	0.337	
Winter	nonRT	She1	None	0.151	0.610	
Winter	RT	She2	Built webs	1.000	NA	
Winter	RT	She3	Fine-grained sediments (e.g., sand, wood)	0.151	0.610	
Winter	RT	She4	Leaves	1.000	0.307	
Winter	nonRL	Vol1	Semivoltine	0.841	0.746	
Winter	nonRL	Vol2	Univoltine	0.016	0.257	yes
Winter	RL	Vol3	Bi- & multi-voltine	0.008	0.257	yes
Winter	nonRT	Life1	Very short (< 1 week)	0.841	0.476	
Winter	nonRT	Life2	Short (1 - 4 week)	0.151	0.914	
Winter	RT	Life3	Long (> 1 month)	0.841	0.257	
Winter	RT	Size1	Small (< 9 mm)	1.000	0.476	
Winter	nonRT	Size2	Medium (9 - 16 mm)	0.690	0.914	
Winter	nonRT	Size3	Large (> 16 mm)	0.310	0.476	
Winter	nonRL	Fly1	Weak	0.690	0.476	
Winter	RL	Fly2	Strong	0.690	0.476	
Winter	nonRT	Arm1	None (soft-bodied forms)	0.548	0.257	
Winter	RT	Arm2	Poor (heavily sclerotized)	1.000	0.171	
Winter	RT	Arm3	Good (well protected)	0.690	0.589	
Winter	RL	Shp1	Streamlined	1.000	0.521	
Winter	nonRL	Shp2	Not streamlined	1.000	0.610	
